# Peripheral anti-inflammatory effects explain the ginsenosides paradox between poor brain distribution and anti-depression efficacy

**DOI:** 10.1186/1742-2094-8-100

**Published:** 2011-08-16

**Authors:** An Kang, Haiping Hao, Xiao Zheng, Yan Liang, Yuan Xie, Tong Xie, Chen Dai, Qijin Zhao, Xiaolan Wu, Lin Xie, Guangji Wang

**Affiliations:** 1 Key Laboratory of Drug Metabolism and Pharmacokinetics, State Key Laboratory of Natural Medicines, China Pharmaceutical University, Nanjing 210009, Jiangsu, China

## Abstract

**Background:**

The effectiveness of ginseng in preventing and treating various central nervous system (CNS) diseases has been widely confirmed. However, ginsenosides, the principal components of ginseng, are characterized by poor accessibility to the brain, and this pharmacokinetic-pharmacological paradox remains poorly explained. Anti-inflammatory approaches are becoming promising therapeutic strategies for depression and other CNS diseases; however, previous studies have focused largely on anti-inflammatory therapies directed at the central nervous system. It is thus of interest to determine whether ginsenosides, characterized by poor brain distribution, are also effective in treating lipopolysaccharide- (LPS) induced depression-like behavior and neuroinflammation.

**Methods:**

In an LPS-induced depression-like behavior model, the antidepressant effects of ginseng total saponins (GTS) were assessed using a forced swimming test, a tail suspension test, and a sucrose preference test. The anti-inflammatory efficacies of GTS in brain, plasma, and LPS-challenged RAW264.7 cells were validated using ELISA and quantitative real-time PCR. Moreover, indoleamine 2,3-dioxygenase (IDO) activity in the periphery and brain were also determined by measuring levels of kynurenine/tryptophan.

**Results:**

GTS significantly attenuated LPS-induced depression-like behavior. Moreover, LPS-induced increases in 5-HT and tryptophane turnover in the brain were significantly reduced by GTS. IDO activities in brain and periphery were also suppressed after pretreatment with GTS. Furthermore, GTS-associated recovery from LPS-induced depression-like behavior was paralleled with reduced mRNA levels for IL-1β, IL-6, TNF-α, and IDO in hippocampus. Poor brain distribution of ginsenosides was confirmed in LPS-challenged mice. GTS treatment significantly decreased production of various proinflammatory cytokines in both LPS-challenged mice and RAW264.7 cells.

**Conclusion:**

This study suggests that the anti-depression efficacy of GTS may be largely attributable to its peripheral anti-inflammatory activity. Our study also strengthens an important notion that peripheral anti-inflammation strategies may be useful in the therapy of inflammation-related depression and possibly other CNS diseases.

## Background

Depression is a worldwide problem for humans due to its relatively high lifetime prevalence and its substantial associated disability [1-3]. Commonly used antidepressants, including selective serotonin (5-HT) reuptake inhibitors (SSRIs) and monoamine oxidase inhibitors (MAOs), are effective [4]; however, their therapeutic effects only manifest in 28%-63% of depressed patients [5]. The classic monoamine hypothesis of depression has been challenged, and alternative therapeutic strategies based on novel understandings of the etiology of depression are urgently needed.

Accumulating evidence reveals a close linkage between inflammation and depression. Depressive symptoms frequently develop in chronically infected patients and in patients with inflammatory bowel disease, chronic kidney disease, or rheumatoid arthritis [6-9]. Moreover, an imbalance between pro-inflammatory cytokines (IL-1β, IL-6, IFN-γ, and TNF-α) and anti-inflammatory cytokines (IL-4 and IL-10) is frequently observed in untreated depressed patients [10,11], and increased levels of pro-inflammatory cytokines have been found to correlate well with severity of depression [12]. Although the detailed molecular mechanisms underlying inflammation-related depression remain unclear, it has been suggested that pro-inflammatory cytokines may affect the catabolism and disposition of various neurotransmitters through activation of IDO [13,14] and/or by up-regulation of the serotonin transporter [15-17]. Inflammation-induced IDO activation results in an increased turnover rate for tryptophan, and subsequent accumulation of neurotoxic metabolites such as kynurenine and quinolinic acid, ultimately leading to the development of depression [18].

Because of the close link between peripheral inflammation and depression, it is reasonable to predict a beneficial effect of anti-inflammatory therapy on depression-like behavior. Indeed, the non-steroidal anti-inflammatory drug (NSAID) celecoxib has been proven effective in attenuating chronic and unpredictable stress-induced depression-like behavior, indicating that NSAIDs may be useful for the therapy of depression [19]. Some clinical trials and open-label studies also have found that some NSAIDs can reduce depressive symptoms, in particular fatigue, and improve quality of life [20]. In view of the fact that these drugs distribute efficiently into the central nervous system, it remains uncertain whether peripheral anti-inflammatory therapy might also be effective in producing neuroprotective effects. This idea is of great potential significance in the development of novel CNS drugs because it is always difficult to balance pharmacokinetic and pharmacological performances during the process of drug design [21].

Ginseng, the root of *Panax ginseng C.A. Meyer*, is frequently and widely used as a tonic herb, and ranks the most widely taken herbal medicine in the world [22]. Saponins, also named ginsenosides, are considered responsible for most of the therapeutic benefits of ginseng. Ginseng total saponins (GTS) have been widely confirmed to be effective in preventing and treating various CNS diseases. GTS exhibit anxiolytic-like effects in an elevated plus-maze model [23,24], and GTS's antidepressant-like effects have been reported [25]. In addition, GTS can improve the outcome of cerebral ischemia [26], attenuate neuroinflammation [27], and prevent learning and memory deficits during aging [28]. On the other hand, previous studies by us and others have shown that brain tissue levels achieved for most of the ginsenosides are very low [29,30], and that these are unlikely to reach the concentrations necessary to elicit neuroprotective effects. Such a pharmacokinetics/pharmacological paradox questions the currently well acknowledged concept that CNS drugs should distribute well into the brain to elicit direct neuroprotective effects. Based on the close link between peripheral inflammation and depression, we hypothesized that the central therapeutic effects of GTS might derive from peripheral anti-inflammation activities. To confirm this hypothesis, we developed a peripheral LPS-induced depression mouse model to determine both anti-depression and anti-inflammatory effects of GTS in the periphery and in the CNS. To further verify our hypothesis, the plasma pharmacokinetics and brain distribution of major GST markers were determined in peripherally LPS-challenged mice.

## Methods

### Drugs and reagents

GTS was purchased from the Department of Natural Medical Chemistry, School of Chemistry, Jilin University (Changchun, China); the contents of the major ginsenosides (Rb_1 _19.1%, Rb_2 _and Rb_3 _13.8%, Rc 12.3%, Rd 9.7%, Re 11.8%, Rf 3.5%, Rg_1 _7.1% and Rh_1 _0.7%) were determined using a validated LC-MS method in our laboratory. LPS (L-3129, 0127:B8) was purchased from Sigma (St Louis, MO, USA).

### Animals and treatment

Male CD-1 mice (10-12 week old, 27-30 g) were purchased from the Animal Centre of China Pharmaceutical University. They were individually housed in polypropylene cages and maintained at controlled temperature (~23°C) and relatively humidity (45-55%) under a normal 12 h light/dark cycle (lights on at 08:00 a.m.) with *ad libitum *access to food and water. Mice were handled 2 min each day for 7 days before experimentation to acclimate them to routine handing. All animal studies were approved by the Animal Ethics Committee of China Pharmaceutical University.

For all studies, LPS solutions were freshly prepared in sterile endotoxin-free saline and administered intraperitoneally. The dose of LPS (0.8 mgkg^-1^) was selected because it could induce an acute sickness response in 4 h and reliable depression-like behavior at 24 h post-LPS challenge [13,31]. GTS, dissolved in saline, was intragastrically administered (200 mg.kg^-1^) once daily for 7 days prior to, and on the same day of, LPS injection. One set of animals was used for behavioral testing, including an open field test, a tail suspension test, and a forced swimming test. In another set of mice, brain samples were collected for analysis of 5-HT, tryptophan, and their metabolites at 24 h post LPS challenge (n = 10). Plasma was also collected for analysis of peripheral IDO activity. In a subsequent study, a sucrose preference test was measured 24 h after injection of saline or LPS (n = 6). Mice were then sacrificed and the hippocampi were immediately collected for analysis of mRNA levels for proinflammatory cytokines (IL-1β, IL-6, and TNF-α) and IDO using quantitative real-time PCR. In the final study, mice were injected *i.p*. with saline or LPS; 4 h later, mice were killed and plasma was collected and stored frozen (-80°) until analyzed for proinflammatory cytokines and corticosterone (n = 8).

### Behavior tests

Sucrose preference, tail suspension, and forced swimming tests were employed for behavior tests. Food intake and body weight changes were also recorded at 22 h post LPS injection.

#### Open field test

The open field test was performed before the formal experiments. The cage was divided into nine virtual quadrants, and locomotor activity was measured by counting the number of line crossings and rearings during a 5-min period. Counting was done by two well-trained observers who were blind to the treatments.

#### Sucrose preference test

The sucrose preference test [32,33] was employed to evaluate anhedonia. Before testing, all mice were acclimated to drinking water and 2% sucrose solution in 50 ml conical tubes for 3 days. On the day of testing, drinking water and 2% sucrose solution were placed in the home cage overnight, followed by food and water deprivation for 2 h prior to the testing. At the end of the testing, fluid content was measured and sucrose preference was calculated using the following equation: Sucrose preference (%) = sucrose intake/(sucrose intake + water intake) × 100.

#### Forced swimming test (FST)

The FST was carried out as previously described [34] with slight modifications. Briefly, mice were placed individually in a clear cylinder (diameter 10 cm, height 25 cm), containing 15 cm of water at 25 ± 1°C. The water was changed between testing sessions. Mice were forced to swim for 6 min, and the immobility time during the last 5 min was manually measured by a blinded observer. Mice were considered immobile when they ceased struggling, remained floating motionless, and only made those movements necessary to keep their head above the water [35].

#### Tail suspension test (TST)

The TST was conducted as previously described [36]. Briefly, mice were suspended by adhesive tape that was positioned about 2.5 cm from the tail tap with the head 40 cm above the floor. The trial was carried out for 6 min and the duration of immobility was manually recorded by two blinded observers during the final 5 min interval of the test. Mice were considered immobile when they hung passively and motionlessly.

### Determination of 5-HT, tryptophan, and their metabolites

Mouse brains (about 100 mg) were first weighed and sonicated in 400 μl of 0.1 M HClO_4_/10 μM ascorbate solution for 1 min, and then the brain samples were centrifuged at 12,000 *g *for 15 min at 4°C. An aliquot of 20 μl of supernatant was injected to a reverse-phase high performance liquid chromatograph (HPLC) equipped with electrochemical detection (Shimadzu, Japan) for the determination of 5-HT and 5-hydroxyindoleacetic acid (5-HIAA). Levels of TRP and KYN were detected by a UV detector at 225 nm (for TRP) and 360 nm (for KYN) wavelengths. Concentrations were expressed as ng/g of wet tissue weight. The turnover rate of 5-HT (5-HIAA/5-HT) and Tryptophan (KYN/TRP) was also calculated [13].

### Plasma corticosterone determination

Quantification of plasma corticosterone was performed using an LC-ESI-MS method based on a previous report [37] with minor modifications. The sensitivity (lower limits of quantification) of the corticosterone assay was 10 ng/ml. Intra- and inter-assay coefficients of variation were less than 10%.

### Cytokine enzyme-linked immunosorbent assays (ELISAs)

IL-1β, IL-6, and TNF-α in plasma were measured using a commercially available ELISA kit (Excell, Shanghai, China). Assays were sensitive with lower limits of quantification at 10 pg/ml for IL-1β, IL-6, and TNF-α; inter- and intra-assay coefficients of variation were less than 10%.

### Quantitative real-time PCR

Total RNA from hippocampal samples was extracted in Trizol reagent (Takara, Dalian, China). All reverse transcriptase reactions were carried out by using a Takara PrimeScript 1st Strand cDNA Synthesis Kit according to the manufacturer's instructions.

Real-time PCR analysis was performed in a Thermal Cycler Dice™ Real Time System (Takara, Japan). The sequences of primers used in this experiment are summarized as follows: IL-1β, sense 5'-CTGTGTCTT TCC CGT GGA CC-3'; antisense 5'- CAG CTC ATA TGG GTC CGA CA-3'; IL-6, sense 5'- CCA GAA ACC GCT ATG AAG TTC CT -3'; antisense 5'- CAC CAG CAT CAG TCC CAA GA -3'; TNF-α, sense 5'- ATC CGC GAC GTG GAA CTG -3'; antisense 5'- CAG CTC ATA TGG GTC CGA CA-3'; IDO, sense 5'-GTA CAT CAC CAT GGC GTA TG-3'; antisense 5'- ACC GCC TGG AGT TCT GGA A -3'; β-actin, sense 5'-TCT GGC ACC ACA CCT TCT A-3'; and antisense 5'-AGG CAT ACA GGG ACA GCA C-3'. The SYBR Green I PCR mix kit (Takara, Japan) was used to quantify gene expression. LightCycler reactions were performed in a total volume of 20 μl based on the manufacturer's instructions. Three replicates were performed for each quantitative PCR run.

The mRNA concentrations of all detected genes were normalized to that of β-actin in each sample (using the delta-delta C_t _method). Results are expressed as fold change relative to the vehicle group (saline-treated mice).

### Pharmacokinetics and brain distribution of GTS in mice

Following intragastric administration of GTS (200 mg/kg, once daily) for 6 days, GTS and LPS were administrated simultaneously on the 7^th ^day. Mice were then sacrificed at indicated time points (3-4 mice per time point), and plasma and brain samples were collected. Samples of brain tissues were rinsed, dried, and weighted, and 100 mg of each brain tissue sample was ultrasonicated in 200 μl physiological saline. After centrifugation at 10000 *g *for 5 min, supernatant were collected and analyzed using a validated LC-MS method with small modifications.

### Cell culture and treatment

#### Raw264.7 cell-based proinflammatory cytokine inhibition assay

The murine macrophage cell line, Raw264.7, was cultured in Dulbecco's modified Eagle's medium supplemented with 2 mM glutamine, penicillin G (100 U/ml), streptomycin (100 μg/ml), and 10% fetal bovine serum, and maintained at 37°C in a humidified incubator containing 5% CO_2_. Cells were pre-treated with 1, 5, 20, or 100 μg/ml GTS for 0.5 h, and then co-incubated with 100 ng/ml of LPS for another 24 h. TNF-α and IL-6 levels, in supernatants collected from GTS-treated cells, were measured with an ELISA kit. Samples were analyzed according to the protocols described above.

#### A549 cell-based IDO-1 inhibitory activity assay

A549 cells were maintained in RPMI-1640 supplemented with 100 U/mL penicillin, 100 mg/ml streptomycin, and 10% fetal bovine serum (Gibco-Invitrogen, USA). Cells were cultured at 37°C with 5% CO_2 _and 95% humidity. For the IDO-1 inhibitory activity assay, A549 cells were seeded in 24-well culture plates with ~80% confluence. hIFN-γ (200 U/ml) and were used to induce IDO expression. After 24 h of induction, test compounds at the indicated concentrations were added and incubated for another 5 h, and then 200 μl of supernatant per well was collected to determine the concentration of kynurenine. IDO activity was expressed as pmol/h.mg protein.

#### RBE4 cell-based LAT-1 inhibition assay

RBE4 cells represent an immortalized rat brain endothelial cell line that expresses high levels of LAT-1 [38]. RBE4 cells were grown on rat tail collagen-coated, 24-well plates or tissue culture flasks in medium consisting of Ham's F10 and α-MEM (1:1, volume/volume), supplemented with fetal bovine serum (Gibco-Invitrogen, USA), 100 U/mL penicillin, and 100 mg/ml streptomycin. For the LAT-1 inhibition assay, cells were equilibrated for 20 min at 37°C in a buffered salt solution before uptake experiments. Uptake was initiated by adding 1 ml of pre-warmed buffer containing 30 μM of KYN and GTS (1-100 μg/ml) or a competitive inhibitor of LAT-1, BCH (Sigma, 400 μM). After incubation for 10 min at 37°C, uptake was terminated by washing the cultures with ice-cold phosphate-buffered saline 3 times. Cell lysates were collected for KYN analysis and protein content determination.

### Statistical analyses

Data are expressed as mean ± SEM for the indicated analyses. Two-way analysis of variance [39] was employed to determine significant main effects and interactions between main factors. When appropriate, differences between groups were evaluated by an F protected t-test. For all of the analyses, a value of p < 0.05 was considered statistically significant.

## Results

### GTS improves LPS-associated anorexia and weight loss

**Figure 1:**
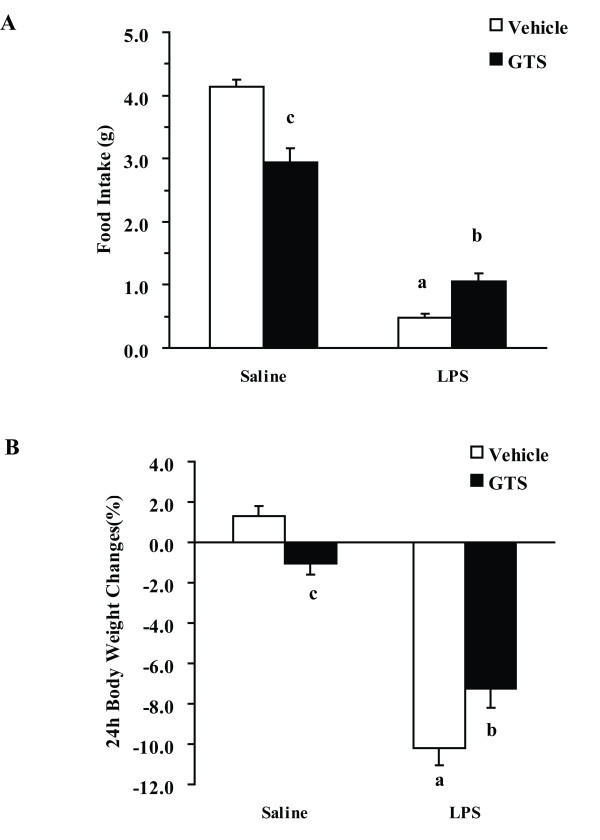
**GTS improves LPS-associated anorexia and weight loss**. Mice were administered orally with GTS at a dosage of 200 mg.kg^-1 ^which was dissolved in saline once daily for 7 days prior to and on the same day of LPS injection. Immediately following the final injection, mice also received either an i.p. injection of sterile endotoxin-free saline or LPS (0.8 mgkg^-1^). The 22-h change in food intake (Figure.1A) and body weight loss (Figure.1B) following LPS administration were measured. The data are expressed as mean ± SEM. For statistical significance, a: p < 0.01 compared with the vehicle/saline group, b: p < 0.05 compared with the vehicle/LPS group. c: p < 0.01 compared with the LPS/GTS group.

As illustrated in Figure 1, GTS × LPS interactions were significantly different for both food intake (F_1,16 _= 19.564, p < 0.01, Figure 1A) and body weight changes (F_1,36 _= 12.804, p < 0.01, Figure 1B) at 24 h post-LPS challenge. Food intake and body weight were significantly reduced in LPS-treated mice as compared with vehicle-treated mice (p < 0.01 for each), whereas mice pretreated with GTS at a dose of 200 mg/kg showed significant protective effect against LPS-associated anorexia (p < 0.05) and weight loss (p < 0.05).

### GTS attenuates LPS-induced depression-like behavior

**Figure 2:**
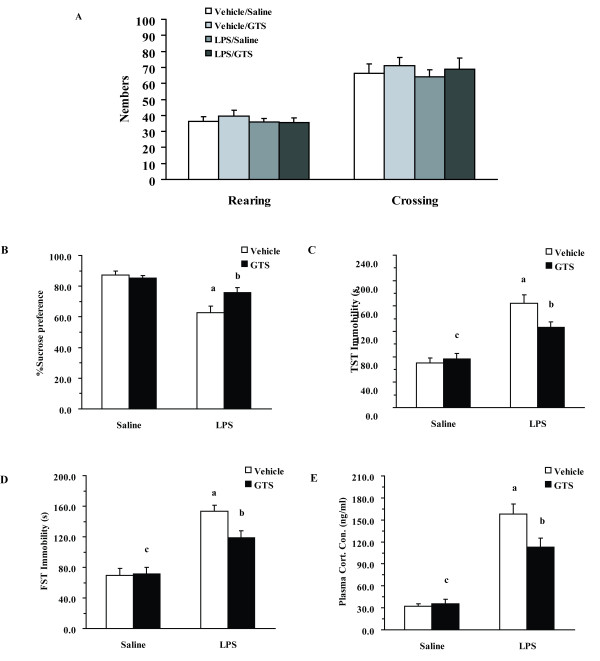
**Effects of GTS on LPS-induced depression-like behavior and corticosterone release**. Mice were treated as in Fig.1. Locomotor activity, expressed as rearings numbers and movement numbers (Figure.2A, n = 10 mice each group), was assessed before the tail suspension test (TST) and the forced swimming test (FST). The sucrose preference test was measured 24 h following administration of LPS (Figure.2B, n = 6 mice each group). Immobility time was assessed during the FST (Figure.2C, n = 10 mice each group) and the TST (Figure.2D, n = 10 mice each group) 22 h and 24 h post-LPS injection. Corticosterone profiles in different groups which were determined 4 h post LPS or saline challenge (Figure.2E, n = 8 mice each group C). The data are expressed as mean ± SEM. For statistical significance, a: p < 0.01 compared with the vehicle/saline group, b: p < 0.05 compared with the vehicle/LPS group. c: p < 0.01 or p < 0.05 compared with the LPS/GTS group.

Locomotor activity tests were performed to exclude the possibility of a nonspecific stimulant action of GTS that could create false-positive results for the FST and TST. As indicated in Figure 2A, there were no significant differences in rearings numbers and movement numbers among different groups.

Sucrose preference index, tail suspension, and forced swimming tests were performed to determine LPS-induced depression-like behavior. As shown in Figure 2B, there was a significant GTS × LPS interaction in sucrose preference (F_1,16 _= 6.102, P < 0.05). LPS-challenged mice exhibited a decreased sucrose preference index as compared with vehicle-challenged mice (P < 0.01), whereas pretreatment with GTS (200 mg/kg) significantly increased the sucrose preference index as compared with mice in the vehicle/LPS group (p < 0.05). Similar results were seen in the tail suspension test and forced swimming test. As shown in Figure 2C and 2D, there was a significant GTS × LPS interaction in both TST (F_1,36 _= 5.239, P < 0.05) and FST (F_1,36 _= 4.737, P < 0.05) for immobility duration. LPS-induced increases in immobility duration in the TST (p < 0.01) and FST (p < 0.01) could be significantly attenuated by GTS pretreatment (p < 0.05 for TST and p < 0.05 for FST).

Corticosterone levels were determined as an indicator of HPA activation. Figure 2E shows the effect of GTS treatment on serum corticosterone levels in LPS-challenged mice. Two-way ANOVA revealed a significant GTS × LPS interaction in serum corticosterone levels (F_1,28 _= 21.933, p < 0.01). LPS caused a significant increase in serum corticosterone levels in mice as compared with saline-treated mice (p < 0.01). GTS treatment decreased serum corticosterone levels in LPS-challenged mice (P < 0.05) as compared with mice in the vehicle/LPS group.

### GTS reduces LPS-induced neuroinflammation and IDO expression

**Figure 3:**
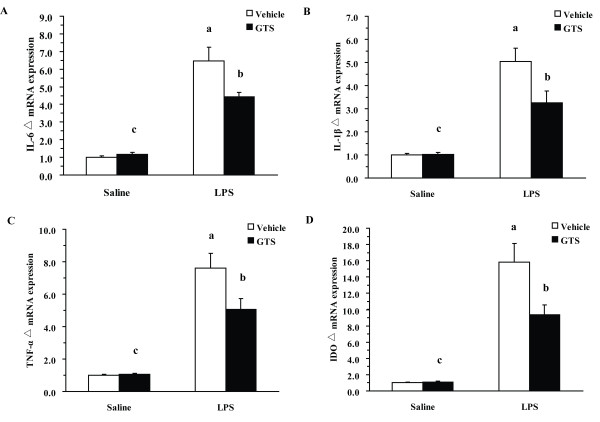
**GTS reduces LPS-induced neuroinflammation and IDO mRNA levels**. Hippocampi were collected 24 h post-LPS challenge and subjected to RNA extraction and Q-RT-PCR. mRNA expression of IL-1β, IL-6, TNF-α, and IDO in hippocampus are presented in Figures 3A, 3B, 3C, and 3D (n = 6 mice each group). The data are expressed as mean ± SEM. For statistical significance, a: p < 0.01 compared with the vehicle/saline group, b: p < 0.05 compared with the vehicle/LPS group. c: p < 0.01 or p < 0.05 compared with the LPS/GTS group.

Neuroinflammation-induced upregulation of IDO expression and activity has been recently verified as an important mechanism of peripheral LPS-induced depression. To investigate whether GTS pretreatment is effective against LPS-induced neuroinflammation, we analyzed proinflammatory cytokines and IDO mRNA expression in hippocampal samples collected 24 h post-LPS challenge. As shown in Figure 3A, B and 3C, there was a significant GTS × LPS interaction for the mRNA expressions of IL-1β, IL-6, and TNF-α (IL-1β: F_1,20 _= 5.603, P < 0.05; IL-6: F_1,20 _= 6.955, p < 0.05, TNF-α: F_1,20 _= 5.505, p < 0.05). Peripheral LPS markedly increased the mRNA expressions of proinflammatory cytokines (IL-1β: P < 0.01; IL-6: p < 0.01, TNF-α: p < 0.01) as compared with the control group. GTS pretreatment at a dose of 200 mg/kg significantly attenuated LPS-induced mRNA upregulation of various proinflammatory cytokines (IL-1β: P < 0.05; IL-6: p < 0.05, TNF-α: p < 0.05).

IDO mRNA levels were determined from the same RNA pool. Figure 3D shows a significant GTS × LPS interaction for mRNA expression of IDO (F_1,20 _= 6.303, p < 0.05). LPS challenge significantly increased IDO mRNA expression in hippocampus (P < 0.01). LPS-induced upregulation of IDO mRNA expression was attenuated by GTS pretreatment at a dose of 200 mg/kg in the hippocampus (p < 0.05), as compared with vehicle/LPS group.

### GTS attenuates LPS-induced metabolic disorders of TRP and 5-HT

TRP, 5-HT, and their metabolites levels are summarized in Table 1. In line with previous reports, the present study shows that brain concentrations of KYN and 5-HIAA are significantly increased after LPS challenge. GTS pretreatment (200 mg/kg) significantly ameliorated LPS-induced alternations of KYN and 5-HIAA levels (P < 0.05 for each). In addition, GTS at a dose of 200 mg/kg reduced the increased turnover rate of TRP and 5-HT in the brain (p < 0.05 for each).

**Table 1:** Effects of GTS administration on the concentrations of TRP, 5-HT, and their metabolites in brain; and on the turnover of TRP and 5-HT (expressed as KYN/TRP and 5-HIAA/5-HT ratios, respectively) (n = 10)

nmol/g brain tissue	Vehicle/Saline	Vehicle/LPS	GTS/Saline	GTS/LPS
**KYN**	0.27 ± 0.03	1.06 ± 0.07^a^	0.29 ± 0.03^c^	0.77 ± 0.04^b^
**TRP**	10.27 ± 0.41	11.75 ± 0.44	10.67 ± 0.38	11.21 ± 0.32
**5-HIAA**	0.95 ± 0.06	1.67 ± 0.13^a^	1.00 ± 0.11^c^	1.34 ± 0.05^b^
**5-HT**	2.70 ± 0.05	2.93 ± 0.21	2.77 ± 0.14	2.93 ± 0.11
**KYN/TRP**	0.026 ± 0.002	0.091 ± 0.006^##^	0.027 ± 0.002^c^	0.067 ± 0.004^b^
**5-HIAA/5-HT**	0.35 ± 0.03	0.57 ± 0.02^##^	0.37 ± 0.03^c^	0.46 ± 0.02*

Dates are expressed as mean ± SEM.

a: p < 0.01 compared with Vehicle/Saline treated mice

b: p < 0.05 compared with Vehicle/LPS treated mice

c: p < 0.05 or p < 0.01 compared with GTS/LPS treated mice.

### GTS reduces LPS-induced peripheral inflammation and kynurenine/tryptophan ratio

**Figure 4:**
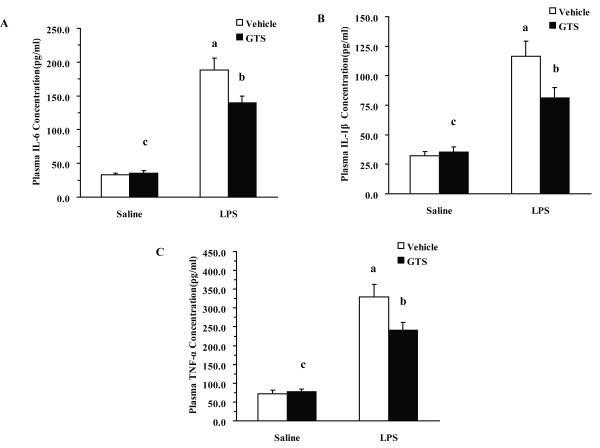
**Effects of GTS on plasma proinflammatory cytokines levels in different groups post LPS or saline challenge**. Mice were treated as in Figure 1. Plasma was collected 4 h after saline or LPS challenge. Cytokines were determined using commercial ELISA kits. The plasma concentrations of IL-1β, IL-6, and TNF-α 4 h post LPS or saline challenge are represented in Figures 4A, 4B, and 4C (n = 8 mice for each group). The data are expressed as mean ± SEM. For statistical significance, a: p < 0.01 compared with the vehicle/saline group, b: p < 0.05 compared with the vehicle/LPS group. c: p < 0.01 or p < 0.05 compared with the LPS/GTS group.

Effects of GTS on regulating plasma proinflammatory cytokines in differently treated mice are shown in Figure 4. Two-way ANOVA showed significant GTS × LPS interactions for the plasma levels of these proinflammatory cytokines among different groups (IL-1β: F_1,28 _= 5.144 p < 0.05; IL-6: F_1,28 _= 6.143, p < 0.05; TNF-α: F_1,28 _= 5.471, p < 0.05). In mice pretreated with vehicle, LPS markedly increased plasma levels of proinflammatory cytokines (IL-1β: P < 0.01; IL-6: p < 0.01, TNF-α: p < 0.01) as compared with the control group at 4 h post-LPS challenge. GTS pretreatment at a dose of 200 mg/kg significantly reduced plasma levels of various proinflammatory cytokines (IL-6: p < 0.05, TNF-α < 0.05, IL-1β < 0.05) as compared with the vehicle/LPS group.

**Figure 5:**
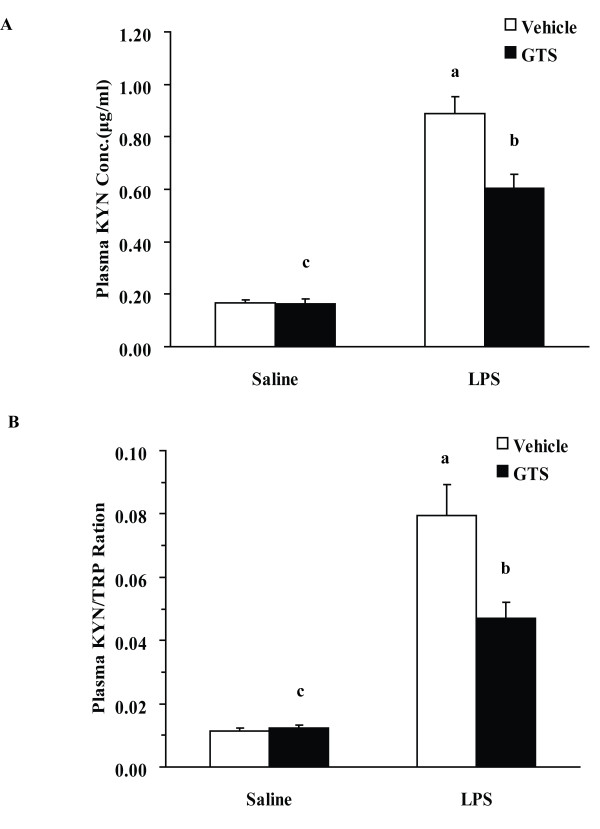
**Effects of GTS on peripheral kynurenine levels and kynurenine/tryptophan ratio**. Kynurenine and tryptophan concentrations were determined by high-performance liquid chromatography in plasma (n = 10 mice for each group). The data are expressed as mean ± SEM. For statistical significance, a: p < 0.01 compared with the vehicle/saline group, b: p < 0.05 compared with the vehicle/LPS group. c: p < 0.01 or p < 0.05 compared with the LPS/GTS group.

Increased peripheral levels of KYN may be an etiogenic factor for the depression-like behavior in this model. Figure 5A shows a significant GTS × LPS interaction in peripheral KYN concentrations (F1,36 = 11.002, p < 0.01). LPS challenge significantly increased peripheral KYN concentrations (P < 0.01). GTS at a dosage of 200 mg/kg could significantly reduce the increased peripheral KYN concentrations in LPS-treated mice (p < 0.01).

As shown in Figure 5B, there was a significant GTS × LPS interaction tested by two-way ANOVA (F1,36 = 8.719, p < 0.01) in KYN/TRP ratios. As expected, we found that the ratio of KYN/TRP was significantly increased in vehicle/LPS treated mice, as compared with control mice. Mice pretreated with GTS at a dose of 200 mg/kg showed a significantly decreased ratio of KYN/TRP (p < 0.05) as compared with mice in the vehicle/LPS group.

### Ginsenosides shows poor brain penetration

**Figure 6:**
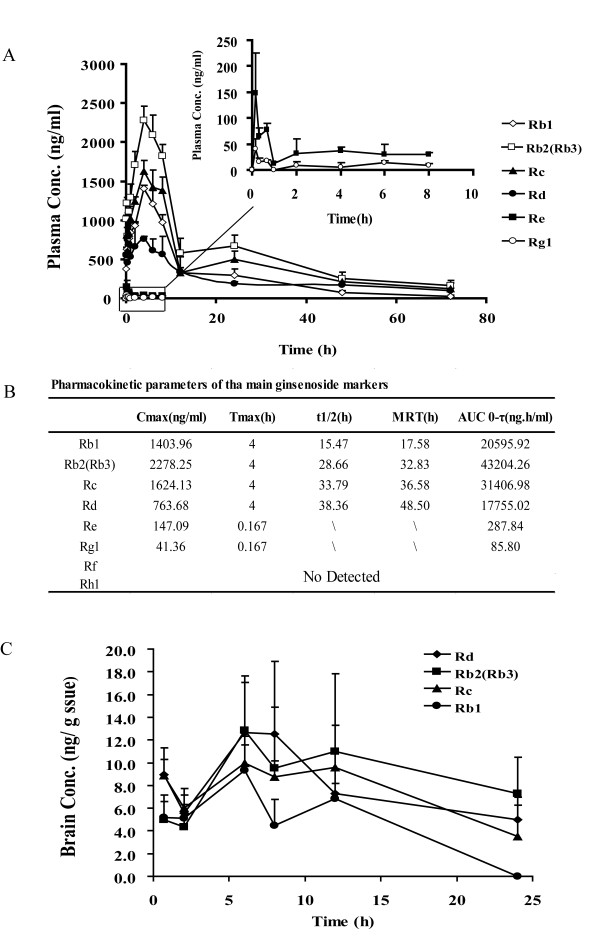
**Pharmacokinetics and brain distribution of GTS in LPS-challenged mice**. GTS was administered by an oral route (200 mg/kg, once daily) for 6 days. On the 7^th ^day, GTS and LPS (i.p.) were administrated simultaneously. Figure.6A represents plasma concentration-time curves for the major ginsenosides (Rb1, Rb2, Rb3, Rc, Rd, Re, and Rg1) in GTS (n = = 3-4 mice per time point). Figure.6B shows the pharmacokinetic parameters of these ginsenosides including maximal concentration (C_max_), time to reach maximal concentration (T_max_), half-life time (T_1/2_), mean retention time (MRT) and area under the curve (AUC). Figure.6C shows a low brain exposure to these ginsenosides, the concentrations of these ginsenosides were all less than 20 ng/g wet tissue. The data are expressed as mean ± SD.

To further confirm our hypothesis that ginsenosides are unlikely to achieve effective concentrations in brain, the pharmacokinetics and brain distributions of major ginsenosides were determined in LPS-challenged mice. As shown in Figure 6A, the plasma profiles of protopanaxadiol-type ginsenoside Rb1, Rb2 (Rb3), Rc, and Rd were successfully characterized after an intragastric administration of GTS (200 mg/kg). The maximum concentrations (C_max_) of the four major ginsenosides reached levels above 1 μg/ml, and their elimination half-life times (t_1/2_) were relatively long (> 20 h), suggesting that the plasma exposure levels of ginsenosides would be sufficient to exert their peripheral anti-inflammatory activities (Figure 6B). However, most of the ginsenosides were undetectable in the brain. Rb1, Rb2 (Rb3), Rc, and Rd were detectable but at extremely low levels of < 20 ng/g wet tissue (Figure 6C).

### GTS inhibits the production of proinflammatory cytokines in LPS-stimulated RAW264.7 cells

**Figure 7:**
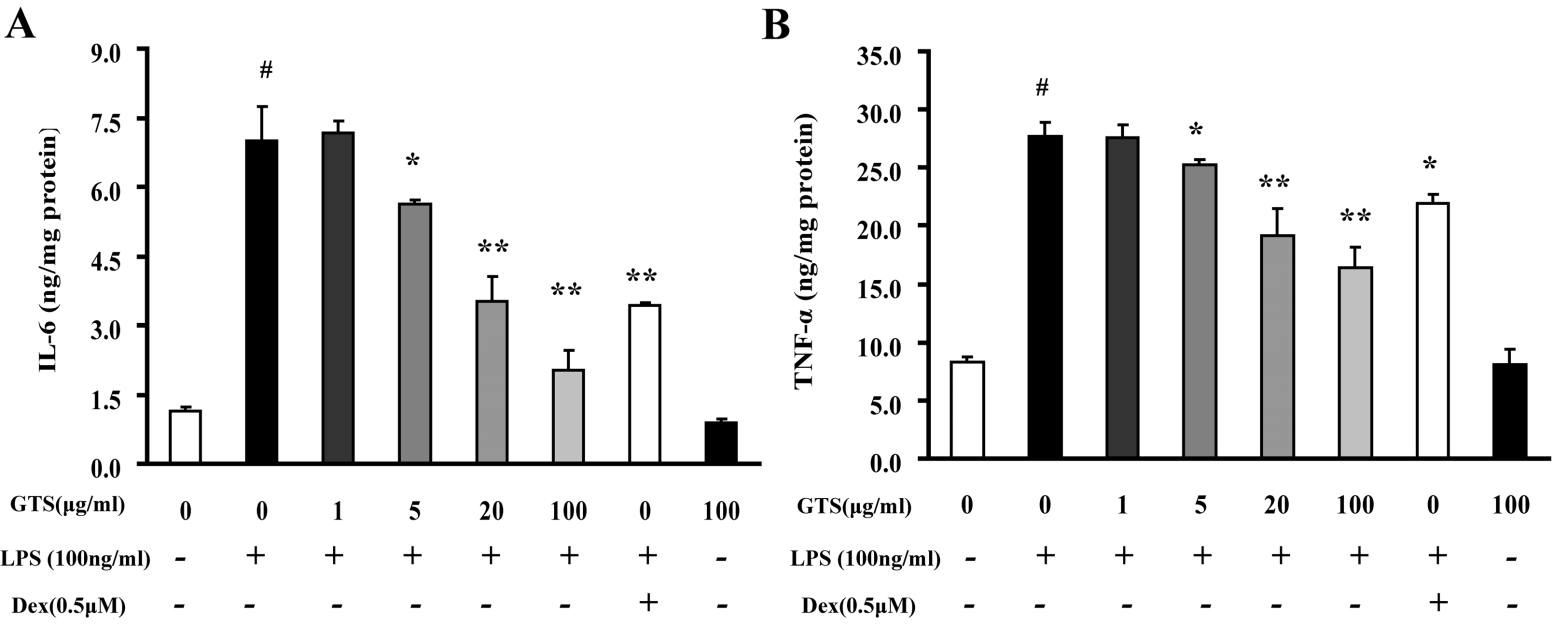
**GTS inhibits production of proinflammatory cytokines in LPS-stimulated RAW264.7 cells**. The murine macrophage cell line, Raw264.7 cells, was used to verify potential peripheral anti-inflammatory effects of GTS. Profiles of IL-6 and TNF-α are shown in Figures 7A and 7B (n = 3). The data are expressed as mean ± SEM. For statistical significance, ## p < 0.01 compared with the no treated RAW264.7 cells, *p < 0.05 **p < 0.01 compared with the LPS (100 ng/ml) treated RAW264.7 cells.

The murine macrophage cell line, Raw264.7 cells, was employed to verify the peripheral anti-inflammatory effects of GTS. As shown in Figure 7A and 7B, GTS had no effect on the basal production of TNF-α and IL-6 in RAW264.7 cells. In contrast, GTS treatment significantly counteracted LPS-stimulated production of TNF-α and IL-6 in RAW264.7 cells in a concentration-dependent manner. Concentrations of major ginsenosides in GTS applied were in a range of 1~100 μg/ml, which is comparable with those detected in mouse plasma.

### GTS has no direct effect on LAT-1 or IDO activity

Because GTS pretreatment was effective in ameliorating LPS-stimulated KYN increases in brain, it was of interest to determine whether or not GTS could directly influence the catabolism and transport of KYN. Presumably, the inhibition of IDO and/or LAT-1 mediated KYN transport across BBB could decrease KYN levels in brain. To address this concern, the effects of GTS on regulating KYN transport and IDO activity were determined in rBE4 cells and A549 cells, respectively.

**Figure 8:**
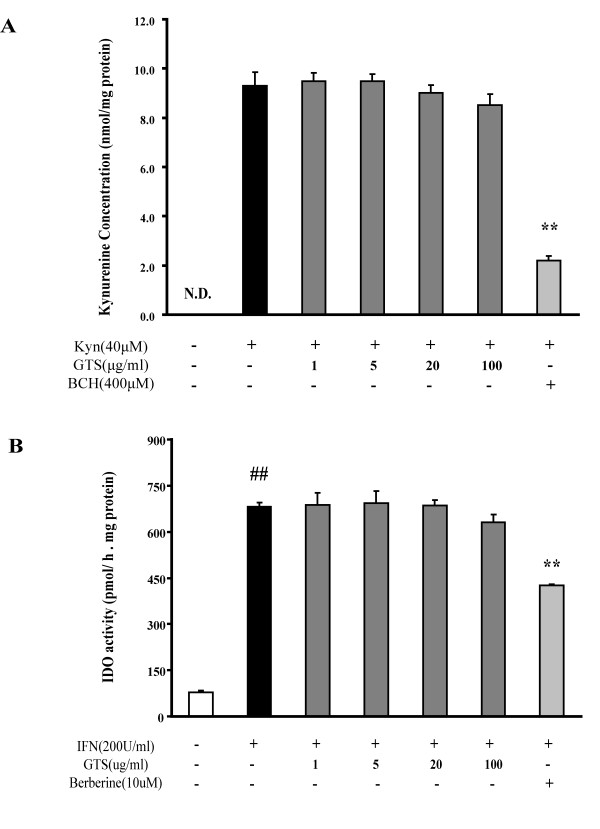
**GTS has no direct effect on LAT-1-mediated kynurenine transport across the BBB and IDO-mediated kynurenine production**. rBE4 cells were used as an *in vitro *model to assess the effects of GTS on KYN transport. A549 cells, stimulated with hIFN-γ, were used as a model to determine GTS effects on IDO activity. Figure.8A shows that GTS has no effect on LAT-1-mediated kynurenine uptake in rBE4 cells (n = 3). Figure.8B reveals that GTS has no effect on IDO-mediated kynurenine production in A549 cells based on an IDO inhibition assay (n = 3). The data are expressed as mean ± SEM. For statistical significance, ## p < 0.01 compared with the no treated A549 cells, **p < 0.01 compared with the kynurenine (40 μM) loaded rBE4 cells or hIFN-γ treated A549 cells.

We used rBE4 cells, an immortalized rat brain endothelium cell expressing high levels of LAT-1, as an *in vitro *model to assess the effect of GTS on KYN uptake. As shown in Figure 8A, GTS had no effect on KYN uptake in rBE4 cells, whereas a competitive inhibitor of LAT-1, BCH (400 μM), significantly decreased the uptake of KYN. Furthermore, mRNA expression of LAT1 and its mediated KYN uptake in rBE4 cells were not influenced by LPS (10 μg/ml) and TNF-α (20 ng/ml) exposure for 24 h (data not show).

A549 cells stimulated with hIFN-γ were used as a model to determine the effects of GTS on regulating IDO activity. Results (Figure 8B) show that GTS had no effect on IDO activity even at a high concentration of 100 μg/ml. In contrast, berberine, a newly identified IDO inhibitor, could significantly decrease production of KYN in A549 cells [40].

## Discussion

Activation of the innate immune system and subsequent release of proinflammatory cytokines may contribute to the development of neuropsychiatric disorders, in particular depression [41], which can be exemplified by the high prevalence of depression in some patients with peripheral inflammatory disorders [20]. Systemic inflammation can influence the CNS through both direct and indirect pathways [41]; proinflammatory cytokines like IL-1β, IL-6, and TNF-α can gain access through relatively permeable areas of the blood-brain barrier [42,43], and activation of the vagal nerve afferent pathway may also account for CNS inflammation originating from the periphery [44]. Emerging evidence suggests the existence of cross-talk between peripheral and central inflammation, which prompted us to hypothesize that a peripheral anti-inflammatory strategy could be useful for the therapy of inflammation-related CNS diseases.

The 'ginseng paradox' between its poor brain distribution and demonstrated neuroprotective effects has been long-standing, but the mysteries behind it remain elusive. Based on the close link between inflammation and depression, we hypothesized that the peripheral anti-inflammation activity of ginsenosides might explain the majority of its anti-depression efficacy. Using a peripheral LPS-challenged depression model, we demonstrate that GTS treatment significantly improves depression-like behaviors, as evidenced from the sucrose preference test, FST, TST, and the determination of plasma corticosterone levels, all of which are typical methods used for characterizing depression [45,46]. In addition, we showed that GTS treatment could significantly inhibit LPS-induced production of multiple proinflammatory cytokines in both the brain and the periphery in mice, and in Raw*264.7 *cell cultures *in vitro*. LPS-induced enhancement of IDO expression/activity, an important mediator between inflammation and depression, was significantly ameliorated by GTS treatment. In line with previous findings [47,48], most of the ginsenosides were found to be almost completely excluded from the brain by the BBB. GTS had no direct effect on IDO activity or KYN transport. Our study strengthens an important concept that peripheral anti-inflammation strategies could be useful for therapy of inflammation-related depression and possibly other CNS diseases.

Anti-inflammation becomes an attractive therapeutic strategy for the treatment of depression, and has indeed been examined at both pre-clinical and clinical levels [20]. Minocycline significantly attenuates LPS-induced neuroinflammation and consequent depression-like behavior in mice [13]. Diclofenac sodium, a non-steroidal anti-inflammatory drug, has been shown to be effective against LPS-induced basic reward behaviors and HPA-axis activation in rats [49]. In view of the fact that such drugs are readily accessible to the CNS, it remains unclear whether a peripheral anti-inflammation strategy could be effective in ameliorating depression-like behavior. The anti-depression effect of minocycline was presumed to originate mainly from its activity on inhibiting LPS-challenged microglial activation, based on the finding that brain but not plasma IL-1β levels were restored toward normal with minocycline treatment [33,50]. Although a previous study showed that GTS could also inhibit activation of microglia in mouse brain inflamed by LPS [27], this effect more likely originated from its peripheral anti-inflammatory activity because GTS would be unlikely to achieve an effective concentration in microglia cells in *vivo*. In the present study, we found that GTS treatment significantly reduced all the tested pro-inflammatory cytokines in both brain and plasma. In contrast to minocycline, most of the ginsenosides contained in GTS are only poorly accessible to brain. Our results indicate that, even in LPS-challenged mice, Rb1, Rb2/Rb3, Rc, and Rd are detectable but at extremely low levels in brain after intragastric administrations of GTS at a dose of 200 mg/kg. The maximum concentrations of such ginsenosides detected in brain were about 20 ng/g tissues, which is far below that needed to exert anti-inflammation effects as observed in various *in vitro *studies (10-100 μM) [27,51,52]. The possibility of brain distribution of some active metabolites of ginsenoside, like compound K, was also excluded by using powerful LC/MS-IT-TOF analysis [53]. Nevertheless, we cannot exclude the possibility that low levels of brain ginsenosides could contribute to the overall anti-depression efficacy of GTS, because the in *vitro *observations may not be directly translatable to *in vivo *outcomes. In spite of this limitation, results obtained from this study strongly suggest that the peripheral anti-inflammatory activities of GTS explain the majority of its effects on neuroinflammation and its consequent anti-depression efficacy.

To further verify the anti-inflammation effects of GTS, a LPS-stimulated murine macrophage cell (Raw264.7) model was used. The results reveal that GTS, at a concentration range of 5-100 μg/ml, can significantly inhibit LPS-induced secretion of TNF-α and IL-6; the effective concentrations observed in this *in vitro *study are comparable with plasma exposure levels of ginsenosides. The maximum concentrations achieved for the four major ginsenosides (Rb1, Rb2/Rb3, Rc, and Rd) reached levels above 1 μg/ml, and the elimination half-life times are longer than 20 h in plasma. These results suggest that systemic exposure levels of ginsenosides could be sufficient to exert peripheral anti-inflammatory activities.

Although the detailed molecular mechanisms connecting inflammation and depression remain largely unclear, it is acknowledged that IDO is an important mediator linking inflammation and depression. Proinflammatory cytokines, in particular IFN-γ and TNF-α, are the main inducers of IDO activation [14,54]. IDO activation results in diverting TRP from synthesis of 5-hydroxytryptophan (5-HTP) and 5-hydroxytryptamine (5-HT) to the generation of TRP metabolites such as quinolinic acid, which is known to be neurotoxic and thereby may lead to depression-like behaviors through both serotonin and glutamate pathways [41,55]. The IDO antagonist, 1-methyltryptophan (1-MT), prevents development of depression-like behaviors in LPS-challenged mice, providing strong evidence for a pivotal role of IDO activation in inflammation-related depression. We found that both hippocampal IDO mRNA levels and plasma IDO activities (KYN/TRP ratio) are significantly reduced by GTS treatment. However, GTS showed no direct inhibitory effect on IDO activity in hIFN-γ-stimulated A549 cell cultures, indicating that the observed IDO-modulating effects of GTS *in vivo *may be secondary to peripheral anti-inflammatory activity.

A recent study showed that exogenous administration of kynurenine to naive mice is able to induce depression-like behavior, suggesting an important role for kynurenine in mediating IDO activation-induced depression [13]. It is important to note that the increased kynurenine levels in brain originate exclusively from the periphery in systemic inflammation models [56]. Upon innate immune system stimulation, the expression and activity of IDO in vascular endothelial cells and macrophages is significantly enhanced, which leads to high plasma levels of kynurenine [13,56], and parallel enhancement of brain kynurenine levels *via *LAT-1-mediated transport [57]. In the uptake experiment performed with rBE4 cells, we found that GTS has no direct effect on kynurenine uptake. In addition, GTS treatment had no effect on exogenous kynurenine administration-induced depression-like behavior (data not shown). Together, our results suggest that GTS pretreatment-induced decreases in kynurenine levels in brain are largely due to reduced peripheral production of kynurenine caused by the peripheral anti-inflammatory activity of GTS.

In view of the fact that the BBB and its constituent cells, including microvascular endothelial cells, pericytes, and microglia, may play an important role in mediating crosstalk between peripheral and central inflammation signals, it would be of great interest in a future study to determine whether GTS could interfere with BBB-mediated crosstalk between periphery and CNS.

**Figure 9:**
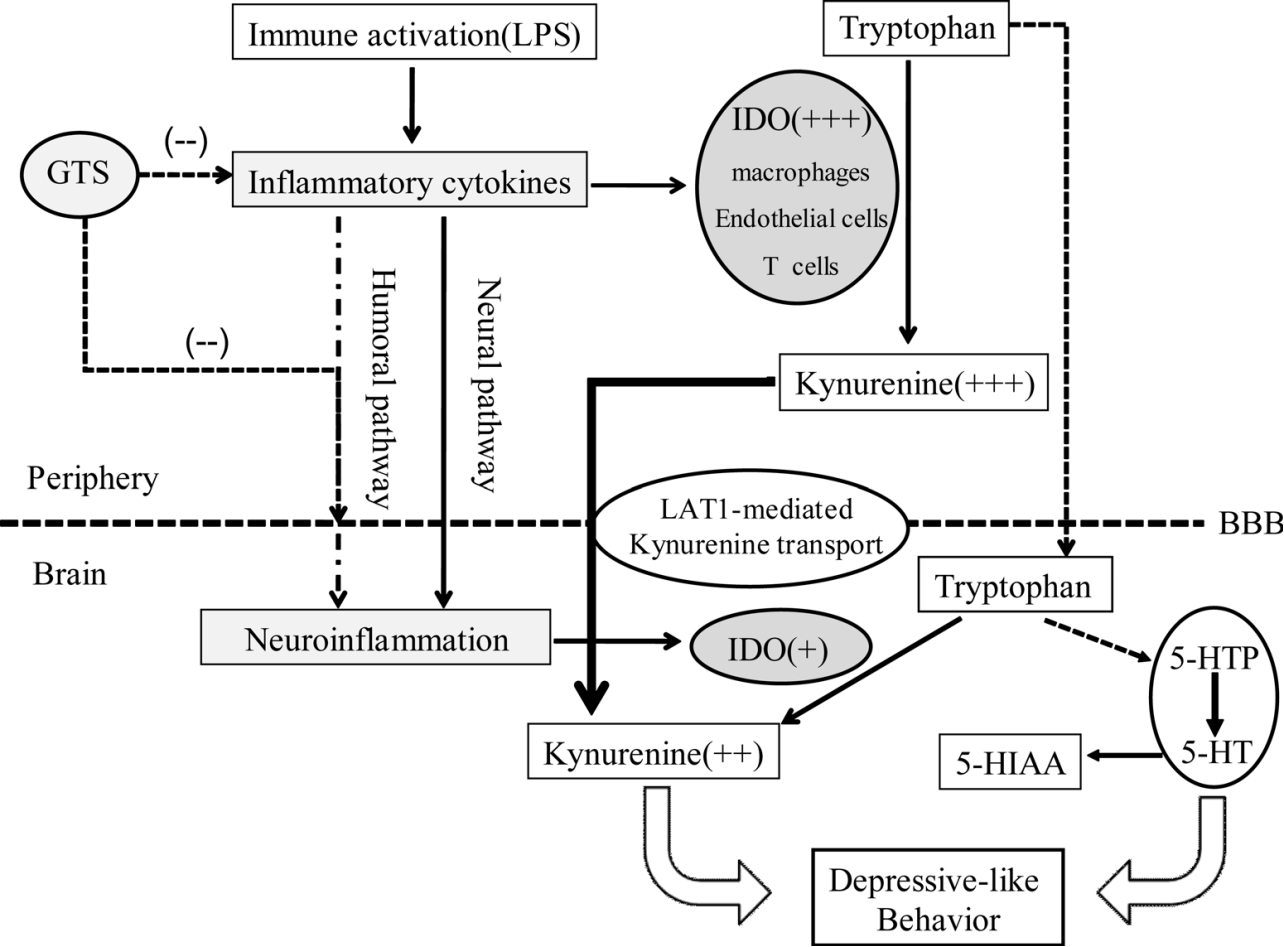
**Proposed anti-depression mechanisms of GTS in the LPS-induced depression model**. Peripheral LPS administration results in production of cytokines, including IL-1β, IL-6, IFN-γ), and TNF-α. These peripheral inflammation signals may be transmitted to the brain through humoral and neural pathways, leading to neuroinflammation. The peripheral and brain cytokines induce transcription and enzymatic activity of IDO, and lead to high levels of kynurenine in periphery and brain. As reported previously, peripheral kynurenine is the main source of increased kynurenine levels in LPS-treated mouse brain through LAT-1-mediated kynurenine active transport across the BBB. Increased kynurenine may metabolize to neurotoxic metabolites like quinolinic acid, thus influencing glutamatergic neurotransmission. Increased IDO activity may also decrease tryptophan availability, thus impacting serotonergic neurotransmission. Inflammation-associated disorder of serotonergic and glutamatergic neurotransmission ultimately induces depression-like behavior. GTS, a well known tonic traditional medicine with poor brain distribution, can improve depression-like behavior in LPS-challenged mice. This seemingly paradoxic concentration-effect relationship could be partially explained by an indirect periphery-to-brain pathway: GTS inhibits LPS-induced peripheral inflammation, thus decreasing neuroinflammation. The inhibition of peripheral inflammation could also partially reverse activation of IDO in the periphery, thus decreasing levels of kynurenine in brain which derive exclusively from periphery in this model, thus ameliorating depressive symptoms.

In conclusion, the present study suggests that GTS could be of therapeutic benefit in inflammation-related depression, and that such an effect largely originates from its peripheral anti-inflammation activities. Figure 9 summarizes the probable mechanisms of GTS effects on ameliorating LPS-induced depression-like behaviors. The present findings not only provide a scientific explanation for the long-standing 'ginseng paradox' between its poor brain distribution and its demonstrated neuroprotective effects, but more importantly, strengthens an important concept that peripheral anti-inflammatory strategies may be useful for the therapy of inflammatory CNS diseases like depression. Such a concept is also supported by the recent finding that etanercept, a recombinant fusion protein of human tumor necrosis factor-α (TNF-α) receptor and immunoglobulin G1, can relieve fatigue and symptoms of depression associated with CNS inflammatory disease or peripheral inflammation [58-60] despite its poor transport across the BBB [61]. More recently, a peripherally restricted inhibitor (URB937) of fatty acid amide hydrolase has been found to be capable of attenuating behavior responses in various rodent models of pain and, meanwhile, avoiding unwanted side effects from activation of brain cannabinoid receptors [62]. In combination with emerging evidence, our results highlight an important notion that the intervention of peripheral targets may be an attractive strategy for the treatment of CNS disorders; such a notion may open up a promising avenue for the rational design of CNS drugs with reduced central side effects.

## Abbreviations

5-HT: serotonin; 5-HIAA: 5-Hydroxyindoleacetic acid; CNS: central nervous system; GTS: ginseng total saponins; HPLC: high performance liquid chromatography; IDO: indoleamine 2,3-dioxygenase; IL: interleukin; KYN: kynurenine; LPS: lipopolysaccharide; NSAIDs: Nonsteroidal anti-inflammatory drugs; TRY: tryptophan, BBB: blood brain barrier.

## Competing interests

The authors declare that they have no competing interests.

## Authors' contributions

HHP and WGJ supervised and designed the study, drafted and revised the manuscript. KA carried out the behavior test, ELISA, and Q-RT-PCR experiments, performed statistical analysis, and drafted and revised the manuscript. XY, ZX, XT, and XL carried out the HPLC, LC-MS experiments. ZQJ and DC carried out the animal treatments. LY and XY designed and supplied the primers used in PCR experiments. WXL, XY, LY, and ZQJ carried out cell cultures. All authors read and approved the final manuscript.

## References

[B1] Moussavi S Chatterji S Verdes E Tandon A Patel V Ustun B Depression, chronic diseases, and decrements in health: results from the World Health Surveys Lancet 2007 370 9590 851 8 10.1016/S0140-6736(07)61415-9 17826170

[B2] Krishnan V Nestler EJ The molecular neurobiology of depression Nature 2008 455 7215 894 902 10.1038/nature07455 18923511 PMC2721780

[B3] Li N Lee B Liu RJ Banasr M Dwyer JM Iwata M Li XY Aghajanian G Duman RS mTOR-dependent synapse formation underlies the rapid antidepressant effects of NMDA antagonists Science 329 5994 959 64 10.1126/science.1190287 PMC3116441 20724638

[B4] Kronenberg S Frisch A Rotberg B Carmel M Apter A Weizman A Pharmacogenetics of selective serotonin reuptake inhibitors in pediatric depression and anxiety Pharmacogenomics 2008 9 11 1725 36 10.2217/14622416.9.11.1725 19018726

[B5] Homberg JR Schubert D Gaspar P New perspectives on the neurodevelopmental effects of SSRIs Trends Pharmacol Sci 2010 31 2 60 5 10.1016/j.tips.2009.11.003 19963284

[B6] Fuller-Thomson E Sulman J Depression and inflammatory bowel disease: findings from two nationally representative Canadian surveys Inflamm Bowel Dis 2006 12 8 697 707 10.1097/00054725-200608000-00005 16917224

[B7] Wolfe F Michaud K Predicting depression in rheumatoid arthritis: the signal importance of pain extent and fatigue, and comorbidity Arthritis Rheum 2009 61 5 667 73 10.1002/art.24428 19404997

[B8] Hedayati SS Minhajuddin AT Afshar M Toto RD Trivedi MH Rush AJ Association between major depressive episodes in patients with chronic kidney disease and initiation of dialysis, hospitalization, or death JAMA 2010 303 19 1946 53 10.1001/jama.2010.619 20483971 PMC3217259

[B9] Moreau M Lestage J Verrier D Mormede C Kelley KW Dantzer R Castanon N Bacille Calmette-Guerin inoculation induces chronic activation of peripheral and brain indoleamine 2,3-dioxygenase in mice J Infect Dis 2005 192 3 537 44 10.1086/431603 15995970

[B10] Thomas AJ Davis S Morris C Jackson E Harrison R O'Brien JT Increase in interleukin-1beta in late-life depression Am J Psychiatry 2005 162 1 175 7 10.1176/appi.ajp.162.1.175 15625217

[B11] Song C Halbreich U Han C Leonard BE Luo H Imbalance between pro- and anti-inflammatory cytokines, and between Th1 and Th2 cytokines in depressed patients: the effect of electroacupuncture or fluoxetine treatment Pharmacopsychiatry 2009 42 5 182 8 10.1055/s-0029-1202263 19724980

[B12] O'Brien SM Scully P Fitzgerald P Scott LV Dinan TG Plasma cytokine profiles in depressed patients who fail to respond to selective serotonin reuptake inhibitor therapy J Psychiatr Res 2007 41 3-4 326 31 10.1016/j.jpsychires.2006.05.013 16870211

[B13] O'Connor JC Lawson MA Andre C Moreau M Lestage J Castanon N Kelley KW Dantzer R Lipopolysaccharide-induced depressive-like behavior is mediated by indoleamine 2,3-dioxygenase activation in mice Mol Psychiatry 2009 14 5 511 22 10.1038/sj.mp.4002148 18195714 PMC2683474

[B14] Wang Y Lawson MA Dantzer R Kelley KW LPS-induced indoleamine 2,3-dioxygenase is regulated in an interferon-gamma-independent manner by a JNK signaling pathway in primary murine microglia Brain Behav Immun 2010 24 2 201 9 10.1016/j.bbi.2009.06.152 19577630 PMC2818058

[B15] Zhu CB Blakely RD Hewlett WA The proinflammatory cytokines interleukin-1beta and tumor necrosis factor-alpha activate serotonin transporters Neuropsychopharmacology 2006 31 10 2121 31 16452991 10.1038/sj.npp.1301029

[B16] Sanchez MM Alagbe O Felger JC Zhang J Graff AE Grand AP Maestripieri D Miller AH Activated p38 MAPK is associated with decreased CSF 5-HIAA and increased maternal rejection during infancy in rhesus monkeys Mol Psychiatry 2007 12 10 895 7 10.1038/sj.mp.4002025 17895923

[B17] Zhu CB Lindler KM Owens AW Daws LC Blakely RD Hewlett WA Interleukin-1 Receptor Activation by Systemic Lipopolysaccharide Induces Behavioral Despair Linked to MAPK Regulation of CNS Serotonin Transporters Neuropsychopharmacology 2010 10.1038/npp.2010.116 PMC3055584 20827273

[B18] Miller AH Norman Cousins Lecture. Mechanisms of cytokine-induced behavioral changes: psychoneuroimmunology at the translational interface Brain Behav Immun 2009 23 2 149 58 10.1016/j.bbi.2008.08.006 18793712 PMC2745948

[B19] Guo JY Li CY Ruan YP Sun M Qi XL Zhao BS Luo F Chronic treatment with celecoxib reverses chronic unpredictable stress-induced depressive-like behavior via reducing cyclooxygenase-2 expression in rat brain Eur J Pharmacol 2009 612 1-3 54 60 10.1016/j.ejphar.2009.03.076 19356723

[B20] Loftis JM Huckans M Morasco BJ Neuroimmune mechanisms of cytokine-induced depression: current theories and novel treatment strategies Neurobiol Dis 2010 37 3 519 33 10.1016/j.nbd.2009.11.015 19944762 PMC2995293

[B21] Gleeson MP Hersey A Montanari D Overington J Probing the links between in vitro potency, ADMET and physicochemical parameters Nat Rev Drug Discov 2011 10 3 197 208 10.1038/nrd3367 21358739 PMC6317702

[B22] Jia L Zhao Y Liang XJ Current evaluation of the millennium phytomedicine- ginseng (II): Collected chemical entities, modern pharmacology, and clinical applications emanated from traditional Chinese medicine Curr Med Chem 2009 16 22 2924 42 10.2174/092986709788803204 19689273 PMC2754208

[B23] Park JH Cha HY Seo JJ Hong JT Han K Oh KW Anxiolytic-like effects of ginseng in the elevated plus-maze model: comparison of red ginseng and sun ginseng Prog Neuropsychopharmacol Biol Psychiatry 2005 29 6 895 900 10.1016/j.pnpbp.2005.04.016 16002200

[B24] Wei XY Yang JY Wang JH Wu CF Anxiolytic effect of saponins from Panax quinquefolium in mice J Ethnopharmacol 2007 111 3 613 8 10.1016/j.jep.2007.01.009 17296279

[B25] Dang H Chen Y Liu X Wang Q Wang L Jia W Wang Y Antidepressant effects of ginseng total saponins in the forced swimming test and chronic mild stress models of depression Prog Neuropsychopharmacol Biol Psychiatry 2009 33 8 1417 24 10.1016/j.pnpbp.2009.07.020 19632285

[B26] Kim YO Kim HJ Kim GS Park HG Lim SJ Seong NS Ham YW Lee SD Jang KH Jung KH Chung JH Kang SA Panax ginseng protects against global ischemia injury in rat hippocampus J Med Food 2009 12 1 71 6 10.1089/jmf.2007.0614 19298198

[B27] Park JS Park EM Kim DH Jung K Jung JS Lee EJ Hyun JW Kang JL Kim HS Anti-inflammatory mechanism of ginseng saponins in activated microglia J Neuroimmunol 2009 209 1-2 40 9 10.1016/j.jneuroim.2009.01.020 19232442

[B28] Zhao H Li Q Pei X Zhang Z Yang R Wang J Li Y Long-term ginsenoside administration prevents memory impairment in aged C57BL/6J mice by up-regulating the synaptic plasticity-related proteins in hippocampus Behav Brain Res 2009 201 2 311 7 10.1016/j.bbr.2009.03.002 19428650

[B29] Xie HT Wang GJ Sun JG Tucker I Zhao XC Xie YY Li H Jiang XL Wang R Xu MJ Wang W High performance liquid chromatographic-mass spectrometric determination of ginsenoside Rg3 and its metabolites in rat plasma using solid-phase extraction for pharmacokinetic studies J Chromatogr B Analyt Technol Biomed Life Sci 2005 818 2 167 73 10.1016/j.jchromb.2004.12.028 15734156

[B30] Li X Wang G Sun J Hao H Xiong Y Yan B Zheng Y Sheng L Pharmacokinetic and absolute bioavailability study of total panax notoginsenoside, a typical multiple constituent traditional chinese medicine (TCM) in rats Biol Pharm Bull 2007 30 5 847 51 10.1248/bpb.30.847 17473424

[B31] Godbout JP Chen J Abraham J Richwine AF Berg BM Kelley KW Johnson RW Exaggerated neuroinflammation and sickness behavior in aged mice following activation of the peripheral innate immune system FASEB J 2005 19 10 1329 31 15919760 10.1096/fj.05-3776fje

[B32] Willner P Towell A Sampson D Sophokleous S Muscat R Reduction of sucrose preference by chronic unpredictable mild stress, and its restoration by a tricyclic antidepressant Psychopharmacology (Berl) 1987 93 3 358 64 10.1007/BF00187257 3124165

[B33] Henry CJ Huang Y Wynne A Hanke M Himler J Bailey MT Sheridan JF Godbout JP Minocycline attenuates lipopolysaccharide (LPS)-induced neuroinflammation, sickness behavior, and anhedonia J Neuroinflammation 2008 5 15 10.1186/1742-2094-5-15 18477398 PMC2412862

[B34] Porsolt RD Bertin A Jalfre M Behavioral despair in mice: a primary screening test for antidepressants Arch Int Pharmacodyn Ther 1977 229 2 327 36 596982

[B35] Pechnick RN Chesnokova VM Kariagina A Price S Bresee CJ Poland RE Reduced immobility in the forced swim test in mice with a targeted deletion of the leukemia inhibitory factor (LIF) gene Neuropsychopharmacology 2004 29 4 770 6 10.1038/sj.npp.1300402 14970834

[B36] Svenningsson P Tzavara ET Qi H Carruthers R Witkin JM Nomikos GG Greengard P Biochemical and behavioral evidence for antidepressant-like effects of 5-HT6 receptor stimulation J Neurosci 2007 27 15 4201 9 10.1523/JNEUROSCI.3110-06.2007 17428998 PMC6672541

[B37] Marwah A Marwah P Lardy H High-performance liquid chromatographic analysis of dehydroepiandrosterone J Chromatogr A 2001 935 1-2 279 96 10.1016/S0021-9673(01)01268-7 11762780

[B38] Gomes P Soares-da-Silva P L-DOPA transport properties in an immortalised cell line of rat capillary cerebral endothelial cells, RBE 4 Brain Res 1999 829 1-2 143 50 10.1016/S0006-8993(99)01387-6 10350540

[B39] Lin EJ Lin S Aljanova A During MJ Herzog H Adult-onset hippocampal-specific neuropeptide Y overexpression confers mild anxiolytic effect in mice Eur Neuropsychopharmacol 2010 20 3 164 75 10.1016/j.euroneuro.2009.08.004 19781916

[B40] Yu CJ Zheng MF Kuang CX Huang WD Yang Q Oren-gedoku-to and its constituents with therapeutic potential in Alzheimer's disease inhibit indoleamine 2, 3-dioxygenase activity in vitro J Alzheimers Dis 2010 22 1 257 66 20847417 10.3233/JAD-2010-100684

[B41] Dantzer R O'Connor JC Freund GG Johnson RW Kelley KW From inflammation to sickness and depression: when the immune system subjugates the brain Nat Rev Neurosci 2008 9 1 46 56 10.1038/nrn2297 18073775 PMC2919277

[B42] Tracey KJ Reflex control of immunity Nat Rev Immunol 2009 9 6 418 28 10.1038/nri2566 19461672 PMC4535331

[B43] Dantzer R Kelley KW Twenty years of research on cytokine-induced sickness behavior Brain Behav Immun 2007 21 2 153 60 10.1016/j.bbi.2006.09.006 17088043 PMC1850954

[B44] Steinman L Nuanced roles of cytokines in three major human brain disorders J Clin Invest 2008 118 11 3557 63 10.1172/JCI36532 18982162 PMC2575716

[B45] Godbout JP Moreau M Lestage J Chen J Sparkman NL OC J Castanon N Kelley KW Dantzer R Johnson RW Aging exacerbates depressive-like behavior in mice in response to activation of the peripheral innate immune system Neuropsychopharmacology 2008 33 10 2341 51 10.1038/sj.npp.1301649 18075491 PMC2907915

[B46] Frenois F Moreau M O'Connor J Lawson M Micon C Lestage J Kelley KW Dantzer R Castanon N Lipopolysaccharide induces delayed FosB/DeltaFosB immunostaining within the mouse extended amygdala, hippocampus and hypothalamus, that parallel the expression of depressive-like behavior Psychoneuroendocrinology 2007 32 5 516 31 10.1016/j.psyneuen.2007.03.005 17482371 PMC1978247

[B47] Liu H Yang J Du F Gao X Ma X Huang Y Xu F Niu W Wang F Mao Y Sun Y Lu T Liu C Zhang B Li C Absorption and disposition of ginsenosides after oral administration of Panax notoginseng extract to rats Drug Metab Dispos 2009 37 12 2290 8 10.1124/dmd.109.029819 19786509

[B48] Gu Y Wang GJ Sun JG Jia YW Wang W Xu MJ Lv T Zheng YT Sai Y Pharmacokinetic characterization of ginsenoside Rh2, an anticancer nutrient from ginseng, in rats and dogs Food Chem Toxicol 2009 47 9 2257 68 10.1016/j.fct.2009.06.013 19524010

[B49] De La Garza R Asnis GM Fabrizio KR Pedrosa E Acute diclofenac treatment attenuates lipopolysaccharide-induced alterations to basic reward behavior and HPA axis activation in rats Psychopharmacology (Berl) 2005 179 2 356 65 10.1007/s00213-004-2053-x 15565429

[B50] Tomas-Camardiel M Rite I Herrera AJ de Pablos RM Cano J Machado A Venero JL Minocycline reduces the lipopolysaccharide-induced inflammatory reaction, peroxynitrite-mediated nitration of proteins, disruption of the blood-brain barrier, and damage in the nigral dopaminergic system Neurobiol Dis 2004 16 1 190 201 10.1016/j.nbd.2004.01.010 15207276

[B51] Jin Y Kotakadi VS Ying L Hofseth AB Cui X Wood PA Windust A Matesic LE Pena EA Chiuzan C Singh NP Nagarkatti M Nagarkatti PS Wargovich MJ Hofseth LJ American ginseng suppresses inflammation and DNA damage associated with mouse colitis Carcinogenesis 2008 29 12 2351 9 10.1093/carcin/bgn211 18802031 PMC2639244

[B52] Ichikawa T Li J Nagarkatti P Nagarkatti M Hofseth LJ Windust A Cui T American ginseng preferentially suppresses STAT/iNOS signaling in activated macrophages J Ethnopharmacol 2009 125 1 145 50 10.1016/j.jep.2009.05.032 19505555 PMC2790430

[B53] Hao H Cui N Wang G Xiang B Liang Y Xu X Zhang H Yang J Zheng C Wu L Gong P Wang W Global detection and identification of nontarget components from herbal preparations by liquid chromatography hybrid ion trap time-of-flight mass spectrometry and a strategy Anal Chem 2008 80 21 8187 94 10.1021/ac801356s 18795791

[B54] Popov A Abdullah Z Wickenhauser C Saric T Driesen J Hanisch FG Domann E Raven EL Dehus O Hermann C Eggle D Debey S Chakraborty T Kronke M Utermohlen O Schultze JL Indoleamine 2,3-dioxygenase-expressing dendritic cells form suppurative granulomas following Listeria monocytogenes infection J Clin Invest 2006 116 12 3160 70 10.1172/JCI28996 17111046 PMC1636691

[B55] Muller N Schwarz MJ The immune-mediated alteration of serotonin and glutamate: towards an integrated view of depression Mol Psychiatry 2007 12 11 988 1000 10.1038/sj.mp.4002006 17457312

[B56] Kita T Morrison PF Heyes MP Markey SP Effects of systemic and central nervous system localized inflammation on the contributions of metabolic precursors to the L-kynurenine and quinolinic acid pools in brain J Neurochem 2002 82 2 258 68 10.1046/j.1471-4159.2002.00955.x 12124427

[B57] Kaper T Looger LL Takanaga H Platten M Steinman L Frommer WB Nanosensor detection of an immunoregulatory tryptophan influx/kynurenine efflux cycle PLoS Biol 2007 5 10 e257 10.1371/journal.pbio.0050257 17896864 PMC1988858

[B58] Tyring S Gottlieb A Papp K Gordon K Leonardi C Wang A Lalla D Woolley M Jahreis A Zitnik R Cella D Krishnan R Etanercept and clinical outcomes, fatigue, and depression in psoriasis: double-blind placebo-controlled randomised phase III trial Lancet 2006 367 9504 29 35 10.1016/S0140-6736(05)67763-X 16399150

[B59] Jiang Y Deacon R Anthony DC Campbell SJ Inhibition of peripheral TNF can block the malaise associated with CNS inflammatory diseases Neurobiol Dis 2008 32 1 125 32 10.1016/j.nbd.2008.06.017 18672064

[B60] Terrando N Monaco C Ma D Foxwell BM Feldmann M Maze M Tumor necrosis factor-alpha triggers a cytokine cascade yielding postoperative cognitive decline Proc Natl Acad Sci USA 2010 107 47 20518 22 10.1073/pnas.1014557107 21041647 PMC2996666

[B61] Hui EK Boado RJ Pardridge WM Tumor necrosis factor receptor-IgG fusion protein for targeted drug delivery across the human blood-brain barrier Mol Pharm 2009 6 5 1536 43 10.1021/mp900103n 19624167

[B62] Clapper JR Moreno-Sanz G Russo R Guijarro A Vacondio F Duranti A Tontini A Sanchini S Sciolino NR Spradley JM Hohmann AG Calignano A Mor M Tarzia G Piomelli D Anandamide suppresses pain initiation through a peripheral endocannabinoid mechanism Nat Neurosci 2010 13 10 1265 70 10.1038/nn.2632 20852626 PMC3260554

